# Epigenetic impact of endocrine-disrupting chemicals on atherosclerosis

**DOI:** 10.1042/EBC20253022

**Published:** 2025-08-26

**Authors:** Ting-An Lin, Changcheng Zhou

**Affiliations:** Division of Biomedical Sciences, School of Medicine, and Environmental Toxicology Graduate Program, University of California, Riverside, CA, U.S.A

**Keywords:** atherosclerosis, cardiovascular disease, endocrinology, epigenetics, microplastics, endocrine-disrupting chenmicals

## Abstract

Atherosclerotic cardiovascular disease (CVD) is the leading cause of mortality and morbidity worldwide. Recent studies have implicated a novel link between exposures to endocrine-disrupting chemicals (EDCs) and CVD. EDCs are a group of persistent compounds that can interfere with the body’s natural hormonal processes, posing significant risks to both environment and human health. However, the impact and underlying mechanisms of EDC exposures on atherosclerosis are poorly understood, making it difficult to conduct rational exposure assessments. EDCs can affect CVD risk through multiple mechanisms, and epigenetic mechanisms are key mechanisms for environmental factor-elicited chronic diseases. Further, EDC-elicited epigenetic alterations may not only affect atherosclerosis development in exposed individuals but also lead to increased CVD risk in their descendants. In this review, we mainly focus on the current understanding of EDC-mediated epigenetic regulation and epigenetic inheritance of CVD. In addition, EDC-carrying microplastics and nanoplastics have emerged as significant environmental pollutants, and humans are ubiquitously exposed to these particles. We also discuss the potential impact of co-exposures of EDCs and small plastic particles on atherosclerosis and CVD.

## Introduction

Atherosclerotic cardiovascular disease (CVD) is the leading cause of mortality and morbidity worldwide mortality [1,2]. Due to the thriving of industrial development, the chemical environment we are exposed to has changed significantly in the past few decades, and exposures to numerous man-made chemicals including endocrine-disrupting chemicals (EDCs) have been implicated in the etiology of CVD [2–10]. EDC is defined as ‘an exogenous agent that interferes with synthesis, secretion, transport, metabolism, binding action, or elimination of natural blood-borne hormones that are present in the body and are responsible for homeostasis, reproduction, and developmental process’ by the U.S. Environmental Protection Agency [11–13]. Some EDCs are present as natural compounds, such as phytoestrogens, but most of the EDCs are synthetic compounds and have been detected in the environment at various concentrations [14]. These compounds can be found in pesticides (e.g., dichloro-diphenyl-trichloroethane [DDT]), incineration by-products (e.g., polychlorinated dibenzodioxins), electrical equipment (e.g., polychlorinated biphenyls [PCBs]), and plastic products (e.g., bisphenol A [BPA]), which can affect environment and human health [14,15].

Mounting evidence demonstrated that EDCs could interfere with hormonal processes in living organisms to elicit adverse consequences [2,11,12,16–20]. The degradation-resistant and stable features of many EDCs often lead to continued accumulation in the environment, which further results in ubiquitous human and animal exposure and unexpected adverse effects [15,21]. Studies of EDCs initially focused on reproductive and developmental toxicity, but mounting evidence demonstrated a strong correlation between EDC exposure and cardiometabolic disease including CVD, obesity, hypertension, and type 2 diabetes [2–8,22–24]. However, the impact of exposure to those EDCs on atherosclerotic CVD risk is poorly understood, making it difficult to conduct rational exposure assessments.

**Figure 1:**
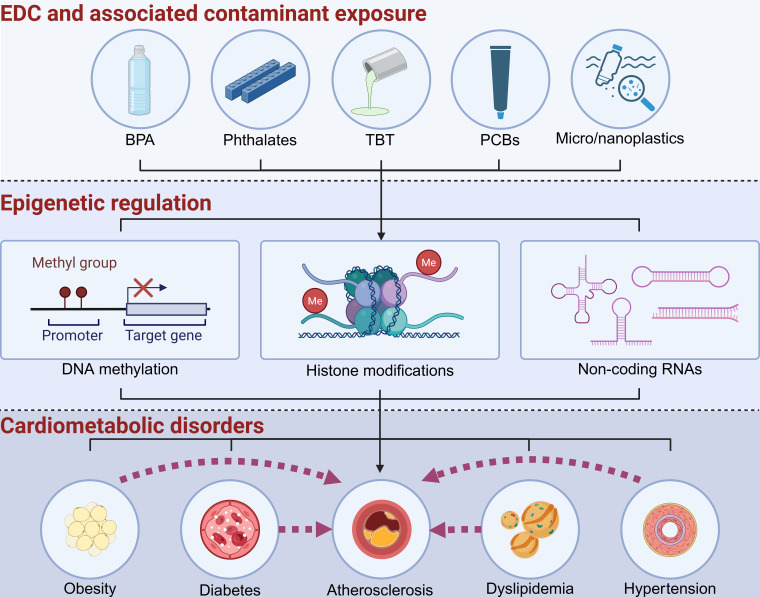
Exposures to EDCs and related environmental contaminants exacerbate atherosclerosis or increase other CVD risk factors through epigenetic regulation. Exposures to EDCs and its related environmental contaminants (e.g., micro- and nanoplastics) contribute to cardiometabolic disorders through epigenetic regulation. The major epigenetic mechanisms include DNA methylation, histone modifications, and noncoding RNAs. BPA, bisphenol A; EDC, endocrine-disrupting chemical; PCBs, polychlorinated biphenyls; TBT, tributyltin. The image was created with BioRender.com.

EDCs may affect CVD risk through multiple mechanisms, and epigenetic mechanisms are key mechanisms for environmental factor-elicited chronic diseases. Epigenetics refers to changes in gene expression and function that do not alter the underlying DNA sequence, serving as a key response mechanism to environmental factors such as diets and EDCs [25,26]. Epigenetics plays a crucial role in linking EDC exposures to the development of various diseases [27–29]. Studies have demonstrated that epigenetic regulation is involved in EDC-induced CVD [2]. This review aims to explore how exposure to EDCs may contribute to the development of CVDs, particularly atherosclerosis, through epigenetic regulations (Figure 1). Further, EDC exposures may also elicit epigenetic inheritance of chronic diseases including obesity, metabolic disorders, and CVD [2,30–33]. Thus, this review focuses on the current understanding of EDC-mediated epigenetic regulation of CVD.

## Effects of EDC exposures on atherosclerotic CVD

Atherosclerosis is a chronic vascular disease characterized by the formation of atherosclerotic plaques, which result from the accumulation of cholesterol, immune cells, and fibroblasts within the walls of arteries [34,35]. The pathogenesis of atherosclerosis is associated with the inflammatory interaction between endothelial cells (ECs), vascular smooth muscle cells (VSMCs), and macrophages [36–40]. Endothelium, the interior layer of blood and lymphatic vessels, first counters with CVD risk factors such as hyperlipidemia, immune reactions, or toxins, leading to endothelial dysfunction and increasing its permeability [41,42]. The infiltrated low-density lipoprotein (LDL) then stays in the intima, the layer underneath endothelium, and forms the fatty streak as a precursor of fibrous plaque, causing the activation of leukocytes and the recruitment of VSMCs [43,44]. The leukocytes, mostly monocytes or macrophages, increase migration and adhesion behavior toward the lesion site, engulf more LDL, and become foam cells [45]. The thickened plaque further triggers the proliferation of VSMCs, depositing fibrous elements such as collagen or derived into macrophages to form a bigger atheroma [46]. The advanced atheroma evolves into plaque rupture and thrombosis, which narrows the blood vessel lumen, leading to ischemic symptoms such as acute ischemic stroke and myocardial infarction (MI) [47,48].

Since EDCs can interfere with various pathways, exposure to these chemicals often leads to inflammatory response, dysregulated lipid metabolism, and insulin resistance, which are all considered CVD risk factors [22,49] (Figure 1). Many EDCs have been shown to have atherogenic effects on humans and animals (Figure 1). For example, circulating PCB levels have been associated with atherosclerotic plaques in humans [9] and animal studies confirmed that exposure to PCBs led to higher circulating cholesterol levels and increased atherosclerosis in animals [50–52]. BPA is a base chemical widely used in polycarbonate plastics manufacturing. Many studies indicate that exposure to BPA may cause adverse health effects in humans [53–55]. Several large and well-controlled cross-sectional and longitudinal studies have found that higher BPA exposure is consistently associated with an increased risk of CVD [4–6]. Other studies have associated BPA exposure with increased coronary atherosclerosis [56], carotid atherosclerosis [7], and peripheral arterial disease [57], a subclinical measure of atherosclerotic vascular disease in humans. Importantly, these associations are independent of traditional CVD risk factors including body mass index, blood pressure, lipid concentrations, and levels of physical activity [4,6]. BPA has been well-known as a weak agonist of the estrogen receptor, and most studies have focused on BPA’s estrogenic effects [58,59]. However, estrogen has been demonstrated to have protective effects against atherosclerosis or CVD in many animal and human studies [60–63]. Thus, the estrogenic effects of BPA may not explain BPA’s atherogenic effects. Interestingly, BPA has also been identified as a potent agonist for human pregnane X receptor (PXR) but not for mouse or rat PXR [64], a nuclear receptor activated by numerous endogenous hormones, dietary steroids, pharmaceutical agents, and xenobiotic chemicals [65–67]. Exposure studies using a PXR-humanized mouse model demonstrated that BPA increased atherosclerosis in atherosclerosis-prone ApoE-deficient mice in a human PXR-dependent manner [68]. Several other studies also reported that BPA can increase atherosclerosis in rabbit models [69,70]. Since the pharmacology of human and rabbit PXR is very similar [67,71], it is plausible that the BPA-mediated rabbit PXR activation also contributes to BPA’s atherogenic effects in those rabbit models.

In addition to BPA, numerous plasticizers such as phthalates are produced in high volume and are used for many products, including toys, food packaging, nutritional supplements, personal cosmetics, cleaning materials, and pharmaceuticals [72,73]. Exposures to various phthalates have also been associated with increased cardiometabolic disease risk and mortality risk in humans, with societal costs ~$39 billion/year or more [16,49,74–81]. For example, circulating levels of phthalates or their metabolites have been associated with atherosclerosis or CVD risk factors such as LDL cholesterol levels [3,7,82]. Several cross-sectional studies demonstrate that urinary phthalate or phthalate metabolite levels are positively associated with carotid intima-media thickness, serum endothelial microparticles and platelet microparticles, and global DNA methylation marker in different human cohorts [83–85]. Some phthalates such as di(2-ethylhexyl) phthalate (DEHP) have been identified as ligands for several nuclear receptors including peroxisome proliferation-activated receptor α (PPARα), PPARγ, and PXR [2,79,86–89]. Activation of those nuclear receptors may lead to the dysregulation of downstream pathways mediating carbohydrate and lipid metabolism. Studies also demonstrated that phthalate exposure caused dysfunction of ECs and VSMCs, key cell types involved in atherosclerosis development [79,90]. For example, DEHP exposure induced cell migration and proliferation by affecting multiple signaling pathways including NF-κB and MAPK pathways in rat VSMCs [90]. Exposure to mono(2-ethylhexyl) phthalate (MEHP), the major metabolite of DEHP, led to increased apoptosis in human ECs [91]. Animal studies also confirmed that DEHP exposure can lead to hyperlipidemia and atherosclerosis development in ApoE^-/-^ mice, which featured increased levels of inflammatory cytokines (e.g., *Tnf-α*, *Mcp-1*) in aorta, and elevated expression of key atherogenic genes (e.g., *Icam-1*, *Vcam-1*) in ECs [92].

While the adverse effects of well-known EDCs such as BPA and DEHP have been well-recognized, recent studies revealed the atherogenic effects of several understudied EDCs. For example, another widely used phthalate, dicyclohexyl phthalate (DCHP), has recently been identified as a ligand for PXR [73]. Animal studies have demonstrated that short-term exposure to DCHP can induce dysregulated lipid homeostasis and hyperlipidemia in wildtype mice [73]. Further, chronic DCHP exposure led to increased atherosclerosis development in LDLR^-/-^ mice in a PXR-dependent manner [93]. In addition to these phthalates, there are many FDA-approved phthalate substitutes including tributyl citrate (TBC), acetyl tributyl citrate (ATBC), and triethyl citrate (TEC) which are considered relatively safe [16,94–98]. Interestingly, several of them, such as TBC and ATBC, have been identified as PXR ligands [89,99]. Animal studies then demonstrated that TBC exposure caused elevated plasma total cholesterol and atherogenic LDL cholesterol levels in mice [89], which may potentially lead to increased atherosclerosis development in exposed populations. Taken together, these epidemiological and toxicological studies have demonstrated that exposure to various EDCs can lead to increased CVD risk, which may be associated with dysregulation of diverse signaling pathways.

## Epigenetic regulation in EDC-induced CVD

**Figure 2:**
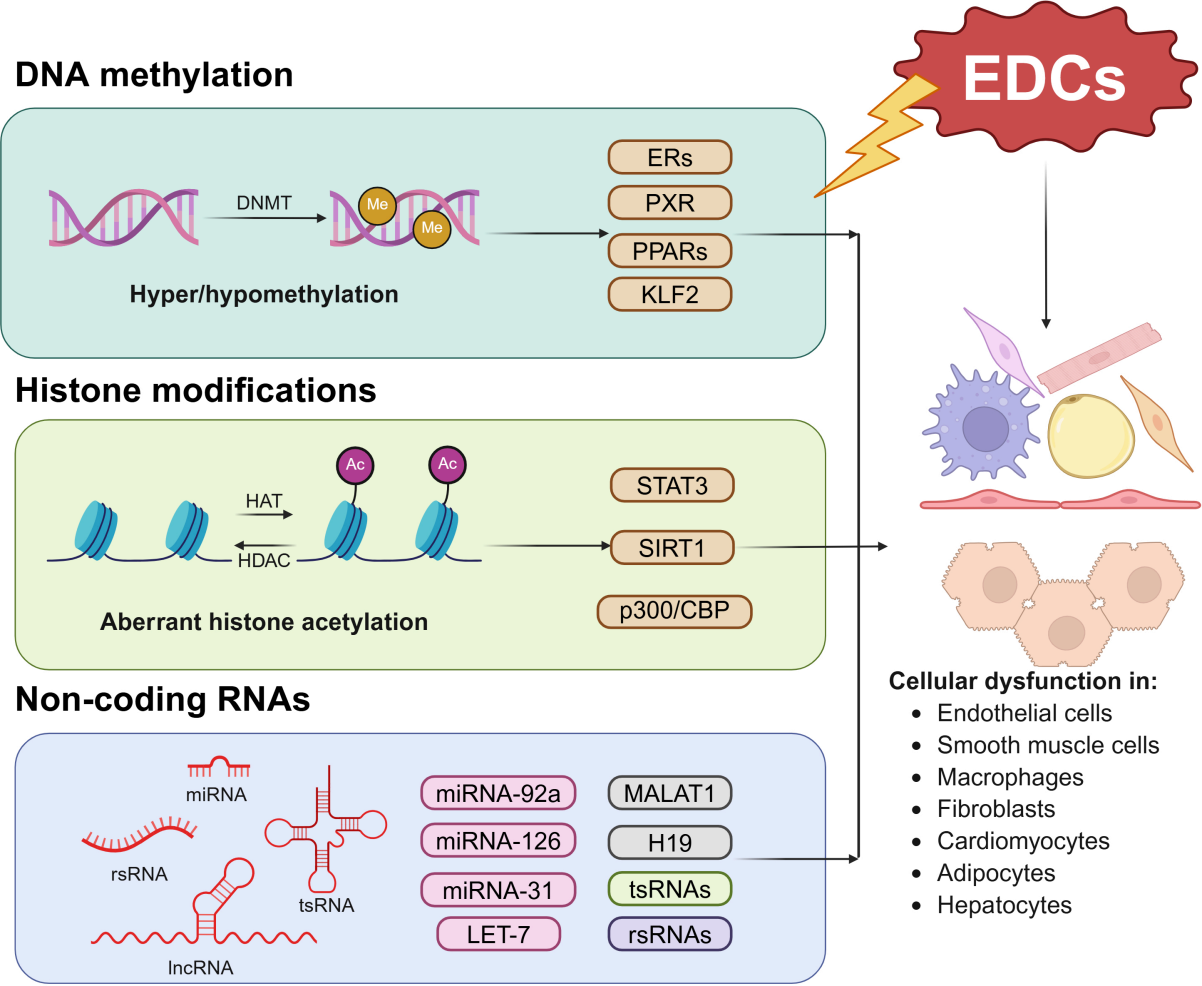
Major epigenetic mechanisms contribute to EDC-elicited atherogenic effects. Three key major epigenetic mechanisms include DNA methylation, histone modifications, and noncoding RNAs contribute to EDC-induced atherogenic effects in multiple cell types. DNMT, DNA methyltransferase; EDC, endocrine-disrupting chemical; HAT, histone acetyltransferases; HDAC, histone deacetylases; lncRNA, long noncoding RNA; miRNA, microRNA; rsRNA, ribosomal RNA-derived small RNA; tsRNA, transfer RNA-derived small RNA. The image was created with BioRender.com.

In contrast with genetic regulation, the epigenome provides dynamic and reversible mediation in gene expression and function, which makes it considered as a mechanism in response to environmental changes [100,101]. Epigenetic regulation plays an important role in the pathophysiology of CVD, and the expression and functions of many CVD-related genes can be modulated by epigenetic regulations [102]. Epigenetic regulation involves three key mechanisms: DNA methylation, histone modification, and noncoding RNAs (ncRNAs) (Figure 2). In this section, we will focus on the impact of EDC exposure on the epigenetic regulation in the context of CVD.

### DNA methylation

DNA methylation refers to adding or removing the methyl groups to usually cytosine residues (e.g., 5-methylcytosine or 5mC). The oxidation of 5mC generates more DNA modifications, including 5-hydroxymethylcytosine (5hmC), 5-formylcytosine (5fC), and 5-carboxylcytosine (5caC), which increase complexity to the landscape of DNA methylation [103]. CpG islands are commonly located in the promoter regions of housekeeping and developmentally important genes, where they typically remain unmethylated to allow active gene transcription under normal conditions. In contrast, aberrant methylation of CpG islands is often linked to transcriptional silencing and is implicated in various diseases [104,105]. Based on the function of methylases, DNA methylation is driven by writing enzymes (e.g., DNA methyltransferases [DNMTs]), erasing enzymes (e.g., ten-eleven translocation family proteins [TETs]), and reading enzymes (e.g., methyl-CpG-binding domain [106] protein, uracil-DNA glycosylase [UNG], and zinc finger protein) [107]. Alternative methylation patterns have been linked to the pathogenesis of CVD [108,109]. For example, overexpression of DNMT1 was associated with the repression of PPARγ activities, leading to increased macrophage inflammatory responses and accelerating the accelerated atherosclerosis development in ApoE^-/-^ mice [110]. Studies also found that TET2-mutant macrophages activate NLRP3 inflammasome to increase IL-1β secretion and promote atherosclerosis development in LDLR^-/-^ mice [111]. Aberrant DNA methylation can affect many genes, including Erα [112,113], ATP-binding cassette A1 (Abca1) [114], and sterol regulatory element-binding proteins (Srebps) [115] that are associated with pathogenesis of CVD including vascular calcification [116], heart failure [117], and MI* [118]*.

EDCs exhibit the ability to induce hyper- or hypomethylation for many CVD-related genes. For example, exposure of pregnant rats to DEHP led to hypermethylation in glucose transporter 4, disrupting the insulin signaling in F1 offspring rats [119]. *In utero* DEHP exposure also raised the global DNA methylation levels in pancreatic β-cells function genes, promoting pancreatic β-cell dysfunction and glucometabolic disorder in the F1 offspring [120]. Epidemiological studies found that urinal MEHP levels were positively associated with subclinical atherosclerosis such as carotid intima-media thickness and global DNA methylation mark 5mdC/dG in a young Taiwanese cohort [83]. Global DNA methylation was positively correlated to apoptotic endothelial microparticles but negatively correlated with adiponectin in serum [85,121]. Although these studies related to DEHP used global DNA methylation assessments, which have limitations in detecting base-specific changes, they provided valuable insight into DEHP-induced aberrant methylation. BPA exposure has also been found to alter methylation in several toxicological and epidemiological studies, which were validated by more precise technology including bisulfite sequencing, pyrosequencing, and methylation-specific PCR (MSP). In utero exposure to BPA adversely affected the hepatic expression of lipogenic genes in high-fat diet (HFD)-fed F1 mice, which was associated with altered DNA methylation [122]. Another study demonstrated that prenatal BPA exposure was positively correlated to methylation level in placental major histocompatibility complex HLA-DRB6, which likely contributed to the exacerbated state of endocrine and metabolic diseases in newborns [123]. Other EDCs, such as arsenic, diethylstilbestrol, DDT, and its metabolites were also found to interfere with chromatin structure through DNA methylation [124–126]. Furthermore, blood-derived epigenome-wide association studies (EWASs) have successfully identified differentially methylated regions in response to exposures such as arsenic, particulate matter (PM_2.5_), and nitrogen oxides (NO_x_) [127,128]. Results from this approach linked EDC exposures to atherosclerosis, highlighting its potential for assessing the impact of EDCs in future research. Collectively, these results suggest that EDC-elicited DNA methylation aberrations may contribute to increased CVD risks in exposed animals or humans.

### Histone modifications

Histone modifications involve post-translational amino acid modifications such as methylation, acetylation, phosphorylation, ubiquitination, and adenylation that remodel chromatin structure and influence the function [129]. The tightened or loosened chromatin structure brings different DNA accessibility to transcription factors, creating the actively transcribed region as euchromatin and inactively transcribed region as heterochromatin [130]. The induction of suppression of DNA accessibility depends on substrates, residues, and sites. For histone methylation, the methylation of histone H3 lysine 4 (H3K4) is often associated with active transcription, whereas the methylation of histone H3 lysine 9 (H3K9) and H3 lysine 27 (H3K27) is associated with inactive transcription [131,132]. In fact, the presence of H3K4me3 alone is not sufficient to predict promoter activity; however, its co-occurrence with H3K27ac is strongly indicative of an active promoter [133,134]. Although both H3K9me3 and H3K27me3 are heterochromatic modifications, H3K9me3 is associated with permanently condensed, constitutively repressed loci, whereas H3K27me3 marks reversible, facultative heterochromatin [135]. Histone acetylation usually occurs at lysine residues and is modulated by histone acetyltransferases (HATs) and histone deacetylases (HDACs) [136]. For example, the silent information regulator sirtuin 1 (SIRT1), a highly conserved NAD^+^-dependent deacetylase, was found to inhibit atherosclerotic plaque formation through regulating the activation of nitric oxide synthase in the ECs of ApoE^-/-^ mice [137,138]. SIRT1 can also down-regulate NF-κB subunit p65 by interfering with the interaction between coactivator p300 and NF-κB, leading to inhibition of atherosclerosis development [139,140].

EDC exposures have been indicated in altering histone modification to have adverse effects in animal studies. For example, DEHP exposure suppressed H3K4me3 levels in oocytes, leading to increased secretion of autophagy proteins and impaired folliculogenesis [141]. The altered histone acetylation was also observed in DEHP-exposed undifferentiated spermatogonia, which was linked to metabolic dysfunction and focal adhesion abnormality in Leydig cells and Sertoli cells [142]. BPA exposure can also enhance histone acetylation in spermatozoa, resulting in chromatin fragmentation in the embryo from male zebrafish [143]. In addition, BPA-impaired spermatozoa showed an increased histone-to-protamine transition, leading to sperm and testes dysfunction [144]. *In utero* exposure to DEHP induced glucometabolic dysfunction in F1 offspring in rats, which is associated with hypermethylation and HDAC2 interaction toward glucose transporter 4 [119]. Paternal exposure to BPA induced histone hyperacetylated landscape in the zebrafish sperm, leading to the overexpression of the histone acetyltransferase and genes involved in cardiogenesis in the progeny [145]. There are limited studies on the impact of EDC exposure-altered histone modification on atherosclerosis development or CVD risk, and future investigations are needed to further explore how EDCs affect histone modifications to influence the pathogenesis of atherosclerosis.

### Noncoding RNAs

Based on the transcription length, ncRNAs are classified into long ncRNAs (lncRNAs, > 200 nt) and small ncRNAs (sncRNAs, < 200  nt). Both lncRNAs and sncRNAs play diverse roles in numerous biological processes including atherosclerosis development [146–150]. For example, several lncRNAs such as MALAT1 and VINAS can regulate atherosclerosis development in vivo [146,149–152]. For sncRNAs, most studies in the CVD field have mainly focused on the functions of microRNAs (miRNAs) [146–148,153]. For example, miRNA-92a is highly expressed in ECs and was found to be induced by shear stress and oxidized LDL in human arterial ECs [154]. Inhibition of miRNA-92a reduced the endothelial inflammation in LDLR^-/-^ mice, which may result from the repression of transcription factor Krüppel-like factor 2 (KLF2) and KLF4 [154,155]. miRNA-146, which targets the 3′ UTR region of *Traf6* to regulate NF-κB activation, has been shown to be associated with atherosclerosis development or CVD in humans [156,157]. Another EC-enriched miRNA, miRNA-126, can negatively modulate vascular inflammation through the NF-κB pathway, ameliorating the progression of atherosclerotic lesion [158,159]. miRNA-31, expressed in ECs and macrophages [160,161], has been shown to contribute to atherosclerosis development [160–162]. In addition to miRNAs, there are other types of sncRNAs including transfer RNA-derived small RNAs (tsRNAs), ribosomal RNA-derived small RNA (rsRNAs), and piwi-interacting RNAs (piRNAs) [163–169]. The functions of these sncRNAs in atherosclerosis remain elusive, but recent studies have identified many tsRNAs and rsRNAs associated with atherosclerosis development in LDLR^-/-^ mice by using a newly developed small RNA sequencing method [170].

EDC exposures have been shown to alter both lncRNAs and sncRNAs to affect cardiometabolic disease risk [171]. For instance, prenatal exposure to BPA can induce lncRNA MALAT1 in the liver, potentially leading to gut microbiota alterations and impaired metabolic pathways in mouse offspring [172]. Another study found that benzyl butyl phthalate disrupts insulin signaling by down-regulating lncRNA H19 and its target microRNAs (miRNA-103/107 and let-7), promoting adipogenesis in C3H10T1/2 stem cells [173]. DEHP exposure led to up-regulation of inflammation-related miRNAs, including miRNA-126–3p, miRNA-181a, and let-7 [174,175]. Paternal exposure to another phthalate, DCHP, can alter sperm tsRNA and rsRNA profiles, potentially contributing to offspring metabolic disorders [176]. While the direct evidence demonstrating EDC-elicited ncRNA changes affecting atherosclerosis or CVD is currently lacking, those studies indicate that the altered ncRNAs by EDC exposure may affect various pathways or risk factors that contribute to the progression of atherosclerosis.

## Epigenetic inheritance of CVD

While the progression of atherosclerosis in humans has been considered to begin during adolescence in humans, atherosclerotic lesions or fatty streaks have been detected in the aorta of fetuses and young children [177,178]. Emerging evidence also indicated that parental environmental factors may affect offspring cardiometabolic health [179–195]. Clinical studies also demonstrated that parents with a history of early-onset CVD may lead to higher CVD risks in offspring [179,181,196]. Most studies in the field have focused on the effects of maternal or *in utero* factors on offspring CVD risk. For example, human studies showed that pregnant mothers with high circulating cholesterol levels may lead to increased fatty streaks in fetal aortas and atherosclerotic lesions in their children [177,178,197,198]. Animal studies confirmed that maternal hypercholesterolemia led to increased atherosclerosis development in the offspring [199–201]. Parental exposure to a range of EDCs can also cause metabolic disorders, and these metabolic disease risks can be transmitted to offspring [183,202–205]. For example, *in utero* or perinatal BPA exposures have been reported to increase metabolic disorders in the offspring [206–210]. Perinatal or maternal exposure to obesogen tributyltin can lead to intergenerational and transgenerational obesity and metabolic disorders [202,204,211,212]. Maternal exposure to BPA elicited exacerbated atherosclerosis in the adult offspring of mice [213]. Multiple epigenetic mechanisms, including histone modifications, DNA methylation, and ncRNAs, have been proposed to contribute to maternal exposure-induced cardiometabolic disease in offspring [31,193–195].

In addition to the well-recognized impact of maternal factors on offspring health, emerging evidence demonstrated that epigenetic inheritance occurs between fathers and their offspring, creating a subsequent wave of research searching for sperm regulatory factors that could relay this transmission of phenotype in a non-DNA sequence-based manner [185,214]. Many studies demonstrated that various paternal exposures could influence offspring cardiometabolic health [184–191,205,215–218]. For example, paternal HFD feeding in mice or rats can lead to increased metabolic dysfunctions in the offspring [184–186,215,218]. Feeding male mice low-protein diets also led to metabolic disorders and decreased vascular function in offspring [188–191]. Paternal high-cholesterol diet (HCD) feeding in LDLR^-/-^ mice elicited sex-specific atherogenic effects in the offspring and led to significantly increased atherosclerosis in F1 female, but not male, LDLR^-/-^ offspring [219]. Paternal exposure to a range of EDCs can also have inter- and trans-generational adverse effects on the metabolic health of their offspring [176,202,205,220]. For example, paternal DCHP exposure led to exacerbated insulin resistance in both male and female F1 offspring [176]. In F2 offspring, paternal DCHP exposure caused sex-specific transgenerational adverse effects and elicited glucose intolerance in female but not male descendants [176]. While various sperm epigenetic factors have been proposed to contribute to intergenerational inheritance of environment-induced phenotypes in mammals [31], sperm sncRNAs, especially tsRNAs and rsRNAs, and their associated RNA modifications may significantly contribute to the intergenerational transmission of paternally acquired cardiometabolic phenotypes [176,214,215,218,219,221]. Indeed, injection of sperm tsRNA/rsRNA-enriched RNA fractions (30–40 nt) from HFD-exposed male mice into normal zygotes generated metabolic disorders in the F1 offspring, while injection of chemically synthesized tsRNAs without RNA modifications cannot induce a similar result [185]. Paternal HCD exposure altered sperm tsRNA/rsRNA landscape in male mice and overexpression of selected sperm tsRNAs and rsRNAs led to increased expression of pro-atherogenic genes in murine embryonic stem cell (mESCs) [219], which may lead to increased atherogenesis in offspring. DCHP exposure can also alter sperm tsRNA/rsRNA landscape [176] but the impact of changed sperm tsRNAs or rsRNAs on mESC gene expression or offspring cardiometabolic health was not investigated.

The influence of parental CVD risk factors on offspring cardiovascular health is still poorly understood, and the underlying mechanisms for the potentially inherited intergenerational or transgenerational effects remain elusive. While the EDC exposure-elicited epigenetic inheritance of obesity and metabolic disorders has been recognized, very few studies have investigated the impact of either maternal or paternal EDC exposures on offspring’s atherosclerosis development. Furthermore, although the transmission of epigenetic information has been consistently observed in laboratory models, the evidence to support the transgenerational effects on human phenotypes is limited, and it is difficult to draw definitive conclusions with current studies [222]. Thus, more studies are needed to investigate the impact and underlying mechanisms of parental EDC exposures on offspring atherosclerosis or cardiovascular health in the future.

## Impact of EDC-carrying microplastics and nanoplastics on CVD

As plastics age and degrade, they become progressively smaller and more highly oxidized on the surface. Microplastics (MPs) and nanoplastics (NPs) are plastic particles with diameters smaller than 1 µm. MPs and NPs are emerging environmental pollutants that have been detected in various environmental media including oceans, soils, atmosphere [223–227] (Figure 1). The widespread presence of plastic particles contributes to food chain contamination, as they are found in drinking water, grains, seafood, meat products, and seasonings [228,229]. Human exposure to MPs and NPs is ubiquitous, and the exposure routes can be through oral intake, inhalation, and dermal contact [230,231]. Plastic particles can be detected in human blood circulatory system [232,233] and many major organs including liver, brain, intestine, stomach, spleen, kidney, lung, testis, placental, and blood vessel [234–237]. Toxicological evidence indicates that MPs/NPs have inflammatory, metabolic, and cell cycle consequences at low exposure concentrations [238–245], leading to increased concerns about their potentially adverse health effects in humans.

Recent studies have also detected MPs and NPs in human arteries and carotid artery plaque [246,247]. Further, patients with carotid artery plaque in which MPs/NPs were detected had significantly increased cardiovascular events than those in whom MPs/NPs were not detected [246]. Another study found that human artery plaques may contain more MPs as compared with the aorta without plaque [247]. These results suggest that exposures to MPs/NPs may affect atherosclerotic plaque formation and increase CVD risk in humans, but the underlying mechanisms remain unknown. MPs and NPs exposures have been reported to affect the functions of macrophages, ECs, and VSMCs [248–251], key cell types regulating atherosclerosis development. For example, MP exposures promoted the pro-inflammatory cytokine expression in macrophages *in vitro* and enhanced macrophage adhesion properties toward ECs [252]. Exposures to NPs may induce human EC leakiness through VE-cadherin signaling pathways [253]. Animal studies confirmed that NP exposures activated phagocytosis of macrophage and also promoted VSMC migration, likely contributing to increased atherosclerosis in ApoE^-/-^ mice [254–256]. Recent studies from our group also demonstrated that MP exposure promotes intimal inflammation and atherosclerosis development in LDLR^-/-^ mice (unpublished observations).

MPs and NPs feature large reaction surfaces, porosity, protein corona, and hydrophobicity, which equip them with a high sorption capacity for organic chemicals [257,258]. Considering the manufacturing of plastic products, plastic particles often appear with EDCs, including BPA and phthalates. MPs and NPs thus may function as EDC carriers and bring those chemicals into human tissues and cells to induce adverse effects [259]. Indeed, studies have shown that EDCs are more easily leached from MPs [238,239] and the synergistic effects of co-exposure of MPs and EDCs have also been implicated in several studies [260,261]. For example, animal studies showed that MPs and DEHP had synergistic effects *in vivo* and co-exposure led to increased kidney proptosis and inflammatory damage in mice [260]. Another study showed that co-exposure of MPs and DEHP impaired the integrity of ovary granulosa cell layer, leading to DNA oxidative damage, cell cycle arrest, and increased necroptosis of mouse ovarian granulosa cells by inducing oxidative stress [261]. While these studies indicated that co-exposure with MPs and EDCs may aggravate the toxic effects *in vivo*, the potential synergistic or additive effects of MPs and EDCs on atherogenesis and CVD have not been investigated.

## Conclusion and perspective

Despite major progress in developing diagnostic techniques and effective treatments, atherosclerotic CVD is still the leading cause of death worldwide. Large-scale human studies have established an important link between exposure to EDCs and CVD. However, how exposure to EDCs influences CVD risk is still poorly understood and continues to hamper rational assessment of the CVD risks of EDC exposures. Epigenetic regulation has been proposed as one of the key mechanisms for environmental factor-elicited chronic diseases. This review discusses the current understanding of EDC-mediated epigenetic regulation of CVD. In addition to summarizing the known effects of EDC exposures on CVD, several major epigenetic mechanisms involved in EDC-induced atherosclerosis of CVD have been discussed. EDC-elicited epigenetic alterations may not only affect atherosclerosis development of exposed individuals but also lead to increased CVD risk in their descendants. However, most research related to intergenerational and transgenerational studies focuses on obesity and metabolic disorders, and it would be interesting to further investigate the detailed epigenetic mechanisms through which EDC exposures influence offspring cardiovascular health. Finally, MPs and NPs have emerged as significant environmental pollutants, and humans are ubiquitously exposed to those particles. Recently, studies started to reveal their potential effects on CVD in humans, but little is known about the underlying mechanisms, including epigenetic mechanisms of MP/NP-induced atherogenic effects. As those particles also carry EDCs, there is also an urgent need to study the synergistic or additive effects of MPs and NPs on EDC-induced atherosclerosis. These studies will contribute to our understanding of EDC-MP/NP interactions in predisposing individuals to CVD.

**Summary**

- Atherosclerosis is the leading cause of death worldwide and exposures to endocrine-disrupting chemicals (EDCs) can increase the risk of developing atherosclerotic cardiovascular disease (CVD).
- EDCs can increase the risk factors for atherosclerosis through epigenetic mechanisms including DNA methylation, histone modification, and noncoding RNAs.
- EDC-elicited epigenetic alterations may also contribute to the increased CVD risk in the offspring of exposed individuals.
- Co-exposure of EDCs and microplastics/nanoplastics may have synergistic or additive effects on CVD.

## Abbreviations

ATBC: acetyl tributyl citrate
CVD: cardiovascular disease
DCHP: dicyclohexyl phthalate
DDT: dichloro-diphenyl-trichloroethane
DEHP: di(2-ethylhexyl) phthalate
DNMTs: DNA methyltransferases
ECs: endothelial cells
EDCs: endocrine-disrupting chemicals
ERα: estrogen receptor α
EWASs: epigenome-wide association studies
HATs: histone acetyltransferases
HDACs: histone deacetylases
LDLR: low-density lipoprotein receptor
MEHP: mono(2-ethylhexyl) phthalate
MI: myocardial infarction
MPs: microplastics
MSP: methylation-specific PCR
NPs: nanoplastics
PCBs: polychlorinated biphenyls
PPAR: peroxisome proliferation-activated receptor
PXR: pregnane X receptor
TBC: tributyl citrate
TBT: tributyltin
TEC: triethyl citrate
TETs: ten-eleven translocation family proteins
VSMCs: vascular smooth muscle cells
5caC: 5-carboxylcytosine
5fC: 5-formylcytosine
5hmC: 5-hydroxymethylcytosine
lncRNAs: long ncRNAs
5mC: 5-methycytosine
miRNAs: microRNAs
ncRNAs: noncoding RNAs
piRNAs: piwi-interacting RNAs
rsRNAs: ribosomal RNA-derived small RNAs
sncRNAs: small ncRNAs
tsRNAs: transfer RNA-derived small RNAs
